# The prevalence of hepatitis B virus infection in Nigerian children prior to vaccine introduction into the National Programme on Immunization schedule

**DOI:** 10.11604/pamj.2016.23.128.8756

**Published:** 2016-03-25

**Authors:** Joanah Ikobah, Henry Okpara, Iwasam Elemi, Yeonun Ogarepe, Ekong Udoh, Emmanuel Ekanem

**Affiliations:** 1 Department of Paediatrics, University of Calabar Teaching Hospital, Calabar, Cross River State, Nigeria; 2 Department of Chemical Pathology, University of Calabar Teaching Hospital, Calabar, Cross River State, Nigeria; 3 Department ofCommunity Medicine, University of Calabar Teaching Hospital, Calabar, Cross River State, Nigeria; 4 Department of Paediatrics, University of Uyo Teaching Hospital, Akwa Ibom State, Nigeria

**Keywords:** Hepatitis B infection, pre-routine immunization, adolescents, Nigeria

## Abstract

**Introduction:**

Hepatitis B virus infection is a major global health problem of public health importance. In a bid to control the infection, the Nigerian government in 2004 introduced hepatitis B vaccine into the National Program on Immunization. There are no studies on the prevalence of hepatitis B in adolescent prior to 2004. The study was aimed at determining the seroprevalence and predictors of viral Hepatitis B in Nigerian children aged 11-19 years.

**Methods:**

A cross sectional analytical study was conducted in July 2014. Multi-staged sampling technique was used to select 749 children from six secondary schools in Calabar, Cross River State, Nigeria. Ethical clearance was obtained from the Cross River State Medical Ethical Committee. A validated structured interviewer administered questionnaire was used to obtain information from participants following parental consent. Blood samples were obtained for qualitative detection of HBsAg using rapid chromatographic immunoassays with test kits from ABON (China) having sensitivity, specificity and accuracy of >99%, 97% and 98.5% respectively. Data was analyzed using SPSS version 20.2.

**Results:**

Nine of the749 students screened were positive for HBsAg giving an overall prevalence of 1.2%. The sex specific prevalence was 0.8% for males and 1.8% for females. After multivariate analysis, age was the predictor of hepatitis B infection (OR 3.92; 95% CI 1.22-12.63; p-value 0.02).

**Conclusion:**

The prevalence of HBV infection was low. Despite the low prevalence, the introduction of the vaccine is justifiable in view of the public health importance of the infection.

## Introduction

Hepatitis B viral infection is a major global health problem with predilection for the liver and is known to commonly lead to chronic infections after the acute infection. The chronic infections increases risk of death from childhood hepatic failure, cirrhosis of the liver and liver cancer [1–4]. The earliest recognition of the public health importance of hepatitis B virus infection is thought to have occurred when it appeared as an adverse event associated with a vaccination campaign [5, 6].

More than 300 million people have chronic liver infections globally and about 600,000 people die annually from acute or chronic complications of hepatitis B infection [5]. The highest prevalence of hepatitis B infection is in sub-Saharan Africa and East Asia [5]. Majority of the people in these regions become infected during childhood and between 5–10% of the adult population is chronically infected [5].

Several studies in Nigerian children have recorded prevalence rates of hepatitis B surface antigen (HBsAg) ranging from 4.1% to 44.7% varying from one locale to another [7–16]. Some of these studies however were hospital based with obvious limitations and different screening methods were used. The pooled prevalence of HBV in Nigeria from studies carried out between 2000 and 2013 is 13.6% and for children was 11.5% [17]. HBV prevalence in Nigeria also varied by the screening method used; the result varied from 12.3% by enzyme-linked immunosorbent assay; 17.5% by immunochromatography; and 13.6% by HBV DNA polymerase chain reaction [17]. HBV infection is thus hyperendemic in Nigeria and may be the highest in sub-saharan Africa [17].

The seroprevalence of HBsAg among children which serves as a proxy for chronic HBV infection is the first indicator that will be affected by an infant vaccination programme and can therefore be used to monitor the programme's impact over a short term [1]. A vaccine against hepatitis B has been available since 1982 [5, 6]. Hepatitis B vaccine is 95% effective in preventing infection and its chronic consequences; it was the first vaccine against a major human cancer [6]. The recombinant vaccines by Pablo DT Valenzuela in 1986 replace the earlier vaccine [5]. In Nigeria, the vaccine was introduced into the National Program on Immunization (NPI) in 2004. It is therefore important to know the prevalence rate of HBV infection in each community before introduction of routine immunization in children.

This work was therefore designed to determine the seroprevalance of Hepatitis B in children who were born before the introduction of hepatitis B vaccine into the NPI schedule in Nigeria. It is hoped that the result will provide the baseline for monitoring of the vaccination program.

## Methods

**Study area**: the study was conducted in Calabar, the capital city of Cross River State, south south geopolitical zone, Nigeria. Calabar has two local government areas (LGA); Calabar Municipality and Calabar South. The population of Calabar Municipality is 183,681 while Calabar South is 191,515. There are a total of 20 public Secondary Schools (14 in Calabar Municipality and 6 in Calabar South) in Calabar. The total student population is 20,993 (12,514 in Calabar Municipality and 8,479 in Calabar South).

**Study design:** the study was a cross sectional analytical study to determine the seroprevalence and predictors of viral hepatitis B in Secondary school children aged 11-19 years.

**Study period:** the study was carried out in July 2014.

**Study population:** the study population consisted of children aged 11 to 19 years who had not been vaccinated against hepatitis B virus.

**Ethical approval:** ethical approval for the conduct of this study was obtained from the Cross River State Health Research Ethics Committee. Clearance was also obtained from Cross River State Ministry of Education and Ministry of Health. Informed consent was obtained from each parent/legal guardian of eligible participants prior to enrolment.

### Sampling technique

Multistage sampling technique was used to recruit subjects for this study. This involved five stages. The first stage was by stratified random sampling technique based on location of schools into Calabar Municipality and Calabar South to select participating students proportionately. Second stage was by simple random sampling to select number of participating schools. In the third stage, each of the six selected schools was stratified based on classes. For each school with six classes (JSS 1, 2, 3, SSS 1, 2 and 3), one sixth of the sample size for the school was equally allocated to each class, for those with classes less than these, the sample size was calculated equally among them.

For the fourth stage, each class was stratified based on streams (that is A, B, C, etc) in the class. The number of children recruited from the class was equally allocated among the streams.

The fifth stage was the final recruitment of a child from a particular stream. Serial numbers of students in the class register was used. From the class register, participants were selected randomly. This ensured that any child in the school could be recruited. Where a subject was to be picked by random selection but was not available in school for any reason or did not satisfy the inclusion criteria, such was dropped and the next was taken.

**Data collection:** data were collected using a structured interviewer administered questionnaire. The following information about the participants were obtained; general characteristics (age, sex), family socioeconomic status based on Oyedeji's classification [18] using parents/guardian's occupation and level of education, social history of the students( sexual exposures, cigarette smoking), numbers of persons in the household, history of jaundice in the household, history of scarification marks and sharing of sharps.

### Laboratory investigations

Blood samples were obtained from all eligible subjects for serological test for HBsAg antibody. Two millitres (2mL) of venous blood was obtained from each participant under aseptic procedure into a properly labeled serial number-tagged clean plain bottle and allowed to clot. Serum was separated and used for the analysis.

Hepatitis B surface antigen (HBsAg) was detected using commercially-available rapid chromatographic immunoassays for the qualitative detection of HbsAg manufactured by ABONTM (Abon Biopharm (Hangzhou) Co., Ltd #19812th Street East, Hangzhou Economic & Technological Development Area, Hangzhou, 310018, P.R. China). The qualitative assays were performed using one-step test strips for detection of HbsAg in serum samples. Test was performed within one hour of specimen collection and separation. The immunochromatographic reaction was allowed to take place within a few minutes and the result read at exactly 15 minutes after.

Only clear, non-haemolyzed serum samples were used. The HBsAg assay has manufacturer-reported diagnostic specificity, sensitivity and accuracy of >99.0%, 97.0% and 98.5% respectively. The results of the test were reported as positive, negative or invalid accordingly. For each invalid test, the test procedure was reviewed and the test repeated with a new strip.

### Statistical analysis

The data obtained was analyzed using statistical package for social sciences (SPSS) version 20.2 Inc. Chicago, Illinois -USA. Categorical and continuous variables were analyzed using Chi-square and Student's t-test respectively. Binary logistic regression analysis was used to control for anticipated confounders. A p-value of 0.05 was considered statistically significant.

## Results

### General characteristics of the study population

A total of 749 children aged between 11 years to 19 years participated in the study. Nine of the participants were positive for HBsAg giving a seroprevalence of 1.2%. Of these, 477 (63.7%) were females and 272 (36.3%) were males giving a ratio of 1.7:1. The age group 14 to 16 years had the highest representation with a total of 384 (51.8%). The mean age of children studied was 14.8 ± 2.0. This is as shown in Table 1.Table 2 shows the bivariate analysis between variables and outcome of HBsAg screening. Age and unsafe traditional practice of scarification mark were statistically significant for positivity to HBV. There was an increasing trend in the age of participants positive for HBs-antigenaemia, this was statistically significant (p = 0.020). Seven out of the nine adolescents were of the low socio-economic class. Table 3 shows the binary logistic regression model for HBsAg positivity, age was the only variable that significantly predicted positivity to HBsAg at both the univariate and bivariate levels (95% CI 1.22-12.63).

**Table 1:** Age group and gender distribution of study population

Variables	Male N = 272 Freq. (%)	Female N = 477 Freq. (%)	Total N = 749 Freq. (%)
**Age(years)**			
11-13	55(7.4)	149(20.1)	204(27.5)
14-16	142(19.1)	242(32.6)	384(51.8)
17-19	72(9.7)	82(11.1)	154(20.8)
**Mean age +/-SD**	15.1 + /-2.1	14.6 + /-2.0	14.8 + /-2.0

**Table 2:** Bivariate analysis between variables and outcome of HBsAg screening

Variable	HbsAg result		Total	χ2 (p- value)
	Positive	Negative		
**Age (years)**				7.837(0,020)
11-13	0(0.0)	204(100.0)	204(100.0)	
14 – 16	4(1.0)	387(99.0)	391(100.0)	
17 - 19	5(3.2)	149(96.8)	154(100.0)	
**Gender Fisher's exact (0.730)**				0.253(0.615)
Male	4(1.5)	268(98.5)	272(100.0)	
Female	5(1.0)	472(99.0)	477(100.0)	
**Number of persons in house**				0.257(0.879)
4	1(0.8)	128(99.2)	129(100.0)	
5- 9	7(1.3)	523(99.2)	530(100.0)	
≥10	1(1.2)	89(98.8)	90(100.0)	
**History of Jaundice in Household Member**				0.579(0.447)
Yes	0(0.0)	45(100.0)	45(100.0)	
No	9(1.3)	695(98.7)	704(100.0)	
**Sexual Exposure**				0.984(0.321)
Yes	1(3.0)	33(97.0)	34(100.0)	
No	8(1.1)	707(98.9)	715(100.0)	
**Smoking of Cigarette**				0.048(0.826)
Yes	0(0.0)	4(100.0)	4(100.0)	
No	9(1.2)	736(98.8)	745(100)	
**Traditional Practice (Scarification mark)**				4.082(0.043)
Yes	3(3.4)	86(96.6)	89(100.0)	
No	6(0.9)	654(99.1)	660(100.0)	
**Socioeconomic status of parents**				Fisher's Exact (0.28)
Low	4(0.9)	425(99.1)	429(100.0)	
Middle	2(1.0)	196(99.0)	198(100.0)	
High	0(0.0)	122(100.0)	122(100.0)	

**Table 3:** Binary logistic regression model of factors that influence seroprevalence of viral hepatitis B

Variables	Odds Ratio	95% CI	p-value
**Age**			
< 14	3.92	1.22 – 12.63	0.02
≥14	1		
**Gender**			
Female	1		
Male	1.05	0.27 – 4.08	0.95
**Number of persons in the household**			
≤14	1.07	0.30 – 3.85	0.92
>14	1		
**History of jaundice in the household**			
No	1		
Yes	2.70	0.0	1.0
**Sexual exposures**			
No	1		
Yes	1.41	0.16 – 12.34	0.76
**Traditional practices (scarification marks)**			
No	1		
Yes	3.14	0.74-13.38	0.12
**Smoking of cigarettes**			
Yes	0.00	0.00	1.0
No	1		
**Parents socioeconomic status**			
Low	0.00	0.00	0.99
Medium/high	1		

## Discussion

The seropositivity of HBsAg in this study was 1.2%. This prevalence is less than most studies carried out in Nigerian children where the prevalence ranged from 4.1% to 44.7% [7–16]. Some of these studies were done in a hospital setting and had fewer sample size compared to the present study. Ugwuja et al [7] carried out their study on adolescent age group in south eastern Nigeria and they had a prevalence of 4.1%. Different screening methods were used for laboratory analysis, this could have accounted for the differences in the prevalence rates [17].

There was a significant association between increasing age and positivity to HBsAg in this study. This is similar to that observed by Abiodun et al [10] in Benin City and Bukbuk et al [13] in Maiduguri in Nigeria. This also supports the assertion that vertical transmission may not play a major role in the spread of HBV infection in Nigeria [19, 20]. In some West African countries, some reports have shown that horizontal transmission may play more role in infection with HBV than vertical transmission [21–23].

In this study, female children had a higher prevalence than their male counterparts though the difference did not reach the level of significance. Donbraye et al [16] working in Osun state, south west, Nigeria also showed higher prevalence in female children than males but the difference similarly did not reach significant level. Bukbuk et al [13] working in northern Nigeria also showed no significant difference between males and females. Other risk factors associated with HBV infection in the study population though not statistically significant included number of persons in the household, unsafe sexual exposure, traditional practice (scarification marks) and low socio-economic status of the family.

This is also in agreement with Al- Faleh et al [24] working in Saudi Arabia who demonstrated that family size and socio-economic status was not significantly associated with HBV positivity in children. Chukwuka et al [14] also working in Ebonyi State, Nigeria showed no significant association between the cultural practice of scarification marks and ear piercing to HBs-antigaenaemia though this also was a risk factor in their study population.

## Conclusion

The study shows a low prevalence of asymptomatic HBV infection among the participants. Although the prevalence is low, the chronicity of the illness and high cost of treatment makes preventive strategies a reasonable option in resource-limited countries. Public health education and vaccination against the virus are therefore advocated for adolescents born in the pre- hepatitis B vaccine era in the Nigeria.

### What is known about this topic

- Hepatitis B is a disease of public health importance which is endemic in many parts of the world
- The highest prevalence of hepatitis B infection is in sub-Saharan Africa and East Asia
- More than 300 million people have chronic liver infections globally and about 600,000 people die annually from acute or chronic complications of hepatitis B infection.

### What this study adds

- This study has provided the prevalence of hepatitis B in adolescents delivered in the pre-vaccination era in Calabar, Cross River State, Nigeria.
- This study shows that though the prevalence of hepatitis B was low in this study population, due to the public health importance of the disease the introduction of vaccination is justified.
- Though not statistically significant, unsafe traditional practice of scarification mark contributed to those positive and the importance of public health importance cannot be over-emphasised.
